# Role of turgor-pressure induced boundary tension in the maintenance of the shoot apical meristem of *Arabidopsis thaliana*

**DOI:** 10.1098/rsif.2023.0173

**Published:** 2023-06-07

**Authors:** Christian Michael, Mikahl Banwarth-Kuhn, Kevin Rodriguez, Calvin-Khang Ta, Amit Roy-Chowdhury, Weitao Chen, G. Venugopala Reddy, Mark Alber

**Affiliations:** ^1^ Interdisciplinary Center for Quantitative Modeling in Biology, University of California, Riverside, CA 92521, USA; ^2^ Department of Mathematics, University of California, Riverside, CA 92521, USA; ^3^ Department of Botany and Plant Sciences, University of California, Riverside, CA 92521, USA; ^4^ Center for Plant Cell Biology, University of California, Riverside, CA 92521, USA; ^5^ Institute for Integrative Genome Biology, University of California, Riverside, CA 92521, USA; ^6^ Department of Computer Science and Engineering, University of California, Riverside, CA 92521, USA; ^7^ Department of Electrical and Computer Engineering, University of California, Riverside, CA 92521, USA; ^8^ Department of Microbiology and Immunology, University of Michigan, Ann Arbor, MI, 48109, USA; ^9^ Department of Mathematics, Cal State East Bay, Hayward, CA 94542, USA

**Keywords:** cell-based model, computational, WUSCHEL, cytokinin, stem cells, plants

## Abstract

In plants, the robust maintenance of tissue structure is crucial to supporting its functionality. The multi-layered shoot apical meristem (SAM) of *Arabidopsis,* containing stem cells*,* is an approximately radially symmetric tissue whose shape and structure is maintained throughout the life of the plant. In this paper, a new biologically calibrated pseudo-three-dimensional (P3D) computational model of a longitudinal section of the SAM is developed. It includes anisotropic expansion and division of cells out of the cross-section plane, as well as representation of tension experienced by the SAM epidermis. Results from the experimentally calibrated P3D model provide new insights into maintenance of the structure of the SAM epidermal cell monolayer under tension and quantify dependence of epidermal and subepidermal cell anisotropy on the amount of tension. Moreover, the model simulations revealed that out-of-plane cell growth is important in offsetting cell crowding and regulating mechanical stresses experienced by tunica cells. Predictive model simulations show that tension-determined cell division plane orientation in the apical corpus may be regulating cell and tissue shape distributions needed for maintaining structure of the wild-type SAM. This suggests that cells' responses to local mechanical cues may serve as a mechanism to regulate cell- and tissue-scale patterning.

## 1. Introduction

In plants, the robust maintenance of tissue structure is crucial to supporting tissue functionality. Unlike animal cells, plant cells have strongly adhered, stiff walls. Since plant cells cannot move relative to one another, the organized tissue growth must be maintained via regulated cell growth and division. Such patterning must be robust to perturbation since a living plant must be able to thrive when exposed to different natural conditions. Even under ideal environmental conditions, a healthy plant must withstand perturbations to tissue patterning due to developmental processes, e.g. surrounding tissue being functional when a primordium begins to bud. Thus, studying the mechanisms involved in and the robustness of cell division and expansion patterning is essential to understanding how plants adapt to changing environments.

**Figure 1.**
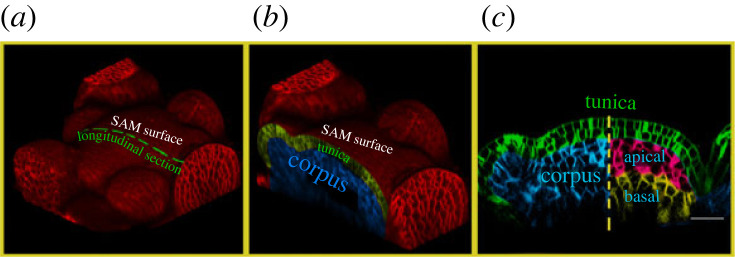
Shape and layered structure of the SAM (*a*) three-dimensional (3D) confocal micrograph of the SAM and surrounding primordia of *Arabidopsis thaliana*. Cell walls are stained in red. A longitudinal sectional contour taken through the SAM apex is illustrated by the green dashed line. (*b*) Longitudinal section of the SAM and adjacent tissue, shown in the context of the 3D tissue. Tunica and corpus are labelled in green and blue, respectively. (*c*) Longitudinal section of the SAM taken from panel (*b*). The SAM is flanked by newly forming primordia. Larger cells below the SAM are expanded, indicating they have begun differentiation. The tunica of the SAM (green cells) comprises two clonally distinct monolayers of cells. The corpus of the SAM is rendered in blue (left), and (right) we consider the corpus as being subdivided into the apical corpus (red) and basal corpus (yellow). Scale bar is 20 µm.

There are multiple examples of tissues whose unique structure is directly related to their mechanical properties on both local and global scale, including organization of epidermal pavement cells [1] and the xylem tissue [2] in plants. In particular, the shoot apical meristem (SAM), which contains a hub of non-differentiating stem cells, maintains a dome shape and specific layer structure at the tip of elongating stems throughout its lifespan (figure 1). The progeny of stem cells in the SAM will eventually become the newly budding primordia in a specific spatio-temporal sequence to produce leaves and flowers. Therefore, the maintenance of the SAM is of critical importance for many plants, and it has been extensively studied in *Arabidopsis thaliana*.

There are a multitude of signals known to influence the shape, size and organization of the SAM. Despite this, we do not know how they influence spatially coordinated direction of cell growth rate, direction of anisotropic expansion, cell division and differentiation [3,4]. Experimentally, it is challenging to determine whether a given hypothesized cell-scale mechanism is at play, as the complex and dynamic interaction between cells within a tissue makes it difficult to determine the emergent behaviour of such a hypothesis. Moreover, many experimental targets for genetic perturbations are upstream and involved in multiple signalling pathways, and these experiments may easily have a combined confounding impact due to modified downstream products.

Many computational modelling approaches have been used to examine specific features of the SAM. The main subjects of study include the epidermal and subepidermal cell layers of the SAM—i.e. cell layers L1 and L2, shown in figure 1. For example, [5] studies how the division patterning of these clonally distinct layers serves to distribute tension isotropically across the SAM surface and minimize the reliance of the tissue upon any single cell for hormone transport. Vertex models have been used to study the L1 of the SAM [6], as well as a plethora of morphogenic phenomena in cell monolayers [7]. However, most existing models of the SAM or similar biological systems were only two-dimensional [6,8] with the highest-resolution level of detail at the cellular scale [9,10].

Recent advancements in three-dimensional microscopy allow for detailed high-resolution three-dimensional spatial data of deep-layer tissue in the SAM. By adapting three-dimensional cell-scale segmentation methods such as spherical harmonic fitting, described in [11], we are able to accurately calibrate detailed multi-scale mechanical models of SAM tissue. Moreover, experiments support the hypothesis that the epidermal L1 and L2 layers are under substantial tension, which may assist in coordinating tissue-scale mechanical cues. Our previous work [12] supports the assumption that in the apical corpus of the SAM, mechanical cues are required for proper division plane patterning, spatial distribution of cells' division plane orientations. In this paper, we study possible impacts of the medial–radial stress experienced by the tunica of the SAM by the surrounding tissue.

To do this, we develop and compare two detailed cell-based two-dimensional (2D) and pseudo-three-dimensional (P3D) models of the SAM. The P3D model incorporates a stochastic, experimentally calibrated representation of cell anisotropy in three dimensions. Both models, which use the subcellular element (SCE) modelling approach from [12], were used to investigate the impact of experimentally calibrated simulated medial–lateral stresses on SAM tissue shape and structure. Calibration of the P3D model was based on image analysis of experimental three-dimensional images of the SAM performed using method of spherical harmonic segmentation [11] (also see electronic supplementary material, SI section S5B for more details).

We found that the inclusion of out-of-plane anisotropically expanded cells allows us to reveal the impact of mechanical stresses on regulating the monolayered structure of the epidermal and subepidermal layers of the SAM. Conversely, we found that without out-of-plane cell expansion, cell–cell crowding dominates the distribution of mechanical stresses experienced by cells in the tunica. Lastly, we found that both 2D and P3D models simulations maintain similar distributions of cell and tissue shapes regardless of the magnitude of mechanical stresses applied to the tunica. We have shown, using model simulations, that this is due to the mechanically driven cell division plane patterning in the apical corpus acting as a regulatory mechanism.

This paper is organized as follows. The Background section contains both biological and modelling contexts for the biological system and modelling methods relevant to this study, respectively. The Model description section provides a justification for the use and applicability of different model components for simulating the longitudinal section of the SAM. This section also contains a detailed presentation of how components are coupled with one another in the 2D and P3D models. Specifically, §3.5 describes the three-dimensional component unique to the P3D model. In the Results section, we discuss the calibration of the model with emphasis on determining ranges of parameters involved in controlling SAM simulated medial–lateral stresses and out-of-plane cell growth components. We then describe computational testing of hypothesized novel biological mechanisms in the Computational model predictions subsection of the Results. In the Discussion, we interpret the results in a general biological context and provide suggestions of potential experiments to test model predictions.

## 2. Background

### 2.1. Biological background

The SAM is a part of tissue located in the growing tip of plants, comprising stem cells that give rise to all above-ground organs. The shape and structure of the SAM are maintained throughout the life of a plant [13,14]. Composing the *Arabidopsis* SAM is the tunica, comprising two clonally distinct layers of epidermal and subepidermal stem cells. The maintenance of the layered structure is crucial for the continued development of the plant, since each layer will produce different cell types; failure to maintain structural arrangement of this tissue will result in the misplacement of organs in the tissue. For example, in mutations in *LOST MERISTEM / HAIRY MERISTEM (HAM)* genes, incorrect maintenance of layered structure results in premature termination of the meristem, which prevents the continued growth of the plant [15].

Below the tunica is the corpus, which does not have layered structure (figure 1*c*), though the growth and division patterns are known to be controlled by WUSCHEL (WUS) and the plant hormone-cytokinin (CK). Because all cells are tightly adhered, the ability for the plant to maintain the shape and structure of the SAM depends mainly on cell-scale anisotropic expansion directions and cell division orientations. Our previous work investigated division patterning in the corpus of the SAM, wherein we found evidence of a qualitative difference in cell division plane placement between the apical and basal regions of the corpus [12].

Experiments have elucidated many of the key factors in the maintenance of the SAM, both from a mechanical and signalling perspective. Techniques such as atomic force microscopy have examined the spatial distribution of the SAM surface via mechanical perturbations [16]. The impact of signalling on the spatial patterning of both the cell and organ scale size and structure of the SAM has been an active area of research [13,17–19]. The critical role of auxin in phyllotaxis patterning and the regulation of auxin itself has been an active area of research as well [20,21]. Multiple levels of control have been shown to spatially regulate the tissue structure of the SAM and signals therein, such as the precise regulation of the WUS protein gradient, cytokinin response and *HAM* [22] in inner cell layers and the transportation of auxin by *PIN* at the SAM periphery [23].

Our previous work [12] sought to understand the role of mechanics on tissue structure by employing two-dimensional SCE models. Advantages of the SCE model are in its ability to mechanistically model heterogeneity within a cell to analyse the emergent tissue-scale impact of such heterogeneity, as in [12,24]. While Banwarth-Kuhn *et al*. established that parallel longitudinal cross-sectional slices of the SAM were similar enough to merit a two-dimensional approach [12], such an approach does not account for effects that may be acting perpendicular to the longitudinal cross-section that was modelled. Though the two-dimensional SCE remains a good choice for investigating mechanical cues that spread across a tissue, the intrinsically three-dimensional nature of the SAM's deep layers makes the impact of three-dimensional organization difficult to study.

### 2.2. Computational modelling background

Many computational models of biomedical and biological systems developed in the form of systems of ordinary or partial differential equation systems, with biological and medical applications spanning from development to epidemiology [25–30], which may be highly computationally intensive. As powerful computer clusters have become more available, the feasibility of more computationally intensive multi-scale modelling methods have expanded greatly to provide description of biological processes at different levels of coarse-grained representation.

One example of a lattice-based model of cell growth, aggregation and migration is the cellular Potts model (CPM) framework, which represents cells as collections of agents on a grid or lattice which interact with one another via complex energy functionals [31–33]. A widely used computational platform based on the CPM is CompuCell3D, which has been used to simulate a plethora of biological phenomena [34,35]. Another class of models are vertex models, typically used to represent locally planar sheets of cells [7,36,37]. The computational platform, VirtualLeaf, simulates plant tissue morphogenesis using an approach which is conceptually similar to the CPM, and bears similarities to the vertex model framework [38–40]. Although the vertex and CPMs are good for modelling many aspects of the cell processes (e.g. proliferation of epithelial sheets [41]; complex adhesion interactions [32,42,43]), it is often difficult to directly represent mechanical properties of a cell. While some classes of vertex models have intuitive energy functionals (e.g. mass-spring models as in [44]), capturing the gradient of stresses along cell walls resulting from differential growth can be challenging in a vertex model framework. Energy functionals may become abstract if not chosen carefully. The SCE model framework is designed to be a high-resolution representation of coarse-grained molecular dynamics and is rooted in representations which are closer to first principles. Though typically computationally intensive, SCE models may directly implement specific mechanical parameters (e.g. elasticity of a material) to obtain simulations that clearly demonstrate the impacts of those parameters. The SCE model framework is particularly well-suited to investigate the impact of intra-cellular and cellular heterogeneity on tissue-scale structuring, especially when cell-scale behaviour (e.g. placement of division planes) is driven by local mechanics (e.g. tension experienced by the cell wall).

SCE models represent cells by a set of heterogeneous, off-lattice nodes interacting with one another through simplified, yet biologically relevant, mechanisms based on potentials and springs of different types. The ability to modify these parameters at the subcellular scale allows for the study of emergence of larger-scale phenomena. An example of this is given in [45], which uses an SCE approach to determine what mechanical properties of the cell are most relevant to mitotic cell rounding observed in the imaginal wing disc in a *Drosophila* embryo. Recently, a SCE framework, PalaCell2D [46], was developed to study morphogenesis using a combination of explicit forces acting on nodes from extracellular sources (e.g. adhesion) and implicit, energy-minimizing forces to simplify the intra-cellular mechanics (e.g. cell area conservation).

Previous modelling studies focused on the interplay between chemical signalling and mechanotransduction phenomena in the SAM [12,24]. In [24], a chemical signalling submodel promoted specific patterns of intra-cellular heterogeneity leading to anisotropic expansion of cells. In our recent work [12], we found evidence that suggested that the cell-scale mechanism controlling the orientations of division planes in the corpus is probably distinct between the apical and basal corpus. Whereas the work in [12] provided insight on the behaviour of the SAM corpus, the monolayer structure of the epidermis of the SAM remained difficult to capture in simulations.

## 3. Model description

This section describes different submodels and how they are linked to one another to provide detailed 2D and P3D models of the 2D longitudinal section of the SAM (figure 1*c*), taking into account tension on the boundary of the model SAM. This model is a 2D representation of a portion of a longitudinal section of a shoot apex taken from *Arabiopsis thaliana.* We use the term *boundary* in this work to refer to the edges of the simulated tissue where, in the true biological system, cells would be connected with the rest of the organism. Coarse-grained forces and other behaviours are often applied to the boundary of a model system to phenomenologically represent interactions with the rest of the organism, as it does not reside in isolation. We would like to indicate that we are not investigating the *SAM* boundary, a morphological structure separating the SAM from forming primordia [43]. Primordia formation is not represented in this work.

**Figure 2.**
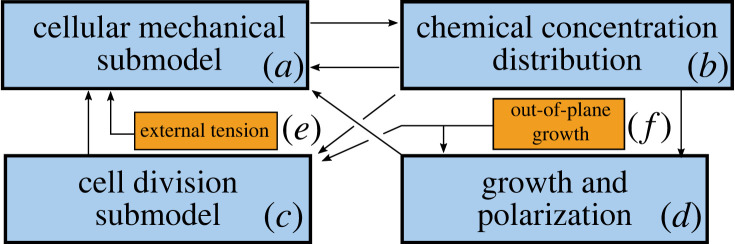
Information flow between the coupled submodels in 2D and P3D models. (*a–d*) Boxes show the interdependencies between the major submodel components. (*a*) The cellular mechanical submodel determines the domain and signal centre for the chemical distribution submodel. (*b*) The impact of WUS and CK are represented by a calibrated spatially dependent approximation of their concentration in cells. CK and WUS levels paramet rize the probabilities of anticlinal versus periclinal expansion as well as growth rate; and the orientation of cell division in the basal corpus. (*c*) The cell division submodel changes the number and position of nodes by adding new cell walls and allowing multiple cell cycles to be represented. (*d*) The growth direction polarization submodel stochastically chooses a preferred anisotropic expansion direction for cells based on their signal concentrations, and cell growth changes the mechanical equilibrium of the mechanical submodel by continually adding new cytoplasm nodes at a WUS-dependent rate. (*e*) Tension applied to the meristem is given by force acting upon the boundary nodes of cells in layers 1 and 2, directed to promote experimentally calibrated curvature. The magnitude of the force is computed in §4.1. (*f*) In the P3D model, out-of-plane growth polarization represents the impact of three-dimensional expansion of cells, as well as how their division is represented.

A diagram of the flow of information between submodels is provided in figure 2 in both the 2D SAM model and the P3D SAM model. The main advantages of these multi-scale models are their detailed biologically calibrated descriptions of cellular growth and division, a detailed implementation of tunica tension boundary conditions and, for the P3D model, the inclusion of cell anisotropic expansion and division both in and out of the model plane. The justification for modelling a specific section of the tissue is given in §3.1.

It was found in [12], by using a 2D model, that cells in the apical corpus of the SAM are likely to divide according to mechanical cues with freely expanding SAM boundary. In this paper, we study, by using a newly developed and calibrated P3D model, the roles of a force distribution applied to the SAM boundary and of out-of-plane growth, on the maintenance of shape and structure of the SAM. Cell growth and division are assumed to be controlled by concentrations of CK and WUS as observed in experiments [12]. Cells are modelled as a heterogeneous collection of nodes representing the cell wall and cytoplasm, which interact with each other through local forces; this is discussed in §3.2.

In the models, cytoplasm nodes are added to each cell at a signal-dependent rate, resulting in turgor pressure increase and leading to addition of new wall nodes to accommodate the build-up of pressure (electronic supplementary material, SI section S1B). Cells are assumed to grow anisotropically entirely within the model domain in the 2D model, or both along the plane or orthogonal to the SAM longitudinal section in the P3D model (see electronic supplementary material, SI section S1C for details on 2D and P3D anisotropic expansion). Cells are assigned a concentration of WUS and CK in the chemical signalling submodel (§3.3) which influences cell growth direction and growth rate (as described in electronic supplementary material, SI sections S1C and S1D). Once cells reach the end of their cell cycle, they divide with a division plane position determined via a mechanism discussed in [12]. Section 3.4 details how these models are coupled together. Lastly, §3.5 describes the representation of out-of-plane cell anisotropic expansion unique to the P3D model.

### 3.1. Experimentally calibrated model of a longitudinal section of the central zone of the shoot apical meristem

At this point, the use of a detailed three-dimensional subcellular element model is prohibitively computationally expensive for large portions of three-dimensional tissue [12], so using a 2D model by leveraging tissue-scale symmetry is critical. Our models represent a longitudinal section of the central region of a SAM as it develops in time. The SAM has been experimentally observed to be approximately radially symmetric in tissue shape, cell size and structure, and signalling distributions (figure 1) with the notable exception being the restructuring leading up to primordium formation. Our previous paper [12] showed that longitudinal sections of the SAM contain information about the shape of the cell the sections were taken from if consideration is restricted to those cells with high aspect ratios. In order to use this assumption in a P3D mechanical model of the SAM, we checked whether this symmetry holds for the distribution of shape features of longitudinal sections of cells.

The tissue-scale approximate radial symmetry is not strictly maintained due to perturbations by developmental processes (e.g. primordium formation). However, a majority of SAM central zones' longitudinal sections look similar regardless of the plane chosen. To support this, we imaged 14 wild-type SAM central zones in three dimensions and, after segmentation and analysis (see electronic supplementary material, section S5 for details), we found that when varying the choice of an axial-basal plane, eight SAMs did not show a significant difference in cell section area distribution, and 13 showed no difference on either cell aspect ratio distribution or directions of anisotropic expansion in cell longitudinal sections. However, even radially symmetric structures may still be affected by forces or cell behaviour directed orthogonally to a longitudinal plane. One such phenomenon, cell expansion and division orthogonal to the longitudinal plane, is represented in the model (see §3.5).

Based on the image analysis, we are able to calibrate 2D and P3D models of the SAM that are capable of matching and investigating cell-scale features. The approximate symmetry of the tissue suggests that conclusions drawn from the longitudinal section provide insight to the true three-dimensional system. Some of the cell-scale mechanical components of the models were calibrated and validated in our previous work [12] by matching simulated and experimental values of cell aspect ratios, orientations of longest axes, and by matching the distribution of cell centroids in simulations to experimental longitudinal sections.

### 3.2. Subcellular element mechanical submodel

Individual cells are modelled as a heterogeneous collection of cellular wall and internal nodes, interacting via potentials representing mechanical forces and moving in two-dimensional space as in [24]. For notational convenience, we keep cell-scale indices as subscripts and subcellular-scale indices as superscripts. Each cell *i* has $N_{i}$ wall nodes denoted as $W_{i}^{j}\;(\;j=1,\ldots ,N_{i})$ and $M_{i}$ cytoplasm nodes denoted as $C_{i}^{j}\;(\;j=1,\ldots ,M_{i})$. We use $W_{i}^{j}$ and $C_{i}^{j}$ to indicate both spatial coordinates of nodes in $R^{2}$ as well as node identity. Moreover, $W_{i}^{j}$ is a neighbour of $W_{i}^{\;j\pm 1}$. To represent the fact that cell walls form a loop, we identify the first and last wall nodes: $W_{i}^{N_{i}+1}=W_{i\;}^{1}$and $W_{i}^{0}=W_{i}^{N_{i}}$. Nearby nodes $W_{k}^{l}$ from adjacent cells (i.e. k≠i) can adhere to node $W_{i}^{j}$. The Langevin equations of motion of individual nodes are as follows:

3.1 $$\begin{array}{rl} \eta_{i}\frac{d}{dt}W_{i}^{j} & =\;-\overset{M_{i}}{\underset{k=1}{\sum }}\nabla E_{\mathrm{Turg}}(W_{i}^{j},C_{i}^{k})\\ & \;-\overset{\;}{\underset{k=j\pm 1}{\sum }}\nabla E_{\mathrm{Ext}}(W_{i}^{k},W_{i}^{j})\\ & \;-\nabla E_{M.F.B.}(W_{i}^{j},W_{i}^{\;j\pm 1})\\ & \;-\overset{\;}{\underset{\mathrm{Cells}\;l}{\sum }}\;\overset{N_{l}}{\underset{k=1}{\sum }}\nabla E_{V.E.D.}(W_{i}^{j},W_{l}^{k})\;\\ & \;-\sum_{\mathrm{Adhesion}\;\mathrm{Partners}\;l}\nabla E_{Adh}(W_{i}^{j},W^{l})+F_{\mathrm{Boundary}} \end{array}$$

and

**Figure 3.**
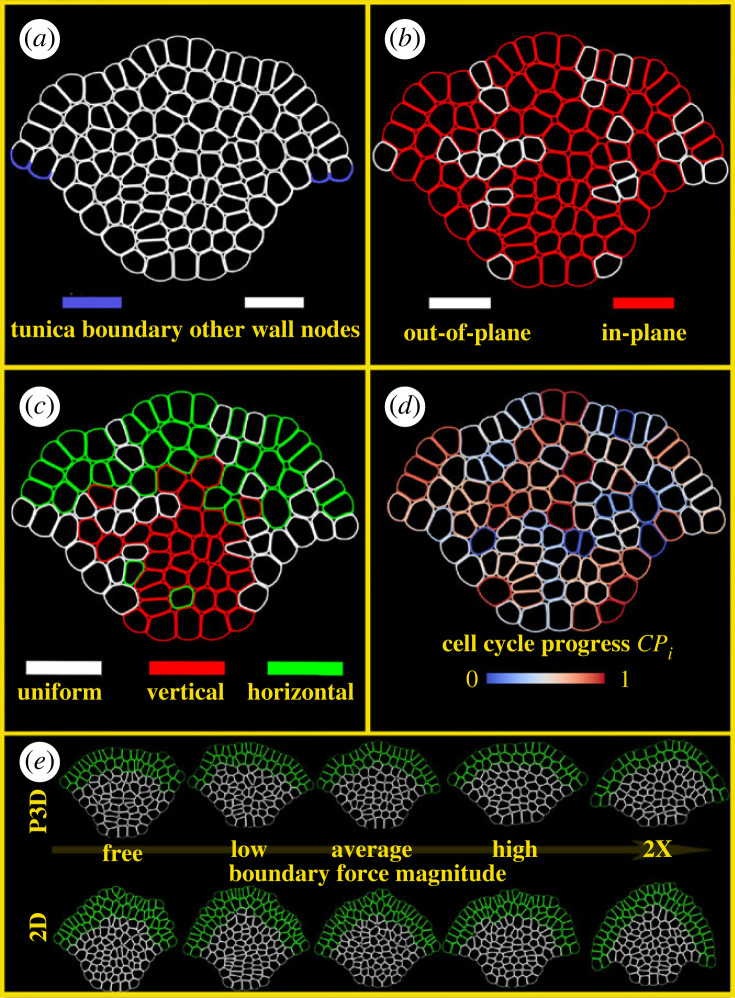
Cell properties during simulations and representative outputs. Four different properties of nodes and cells are indicated by colour mapping. All panels depict different features for the same simulated P3D SAM during a single time step. (*a*) Boundary nodes are shown in blue. Once the direction for $F_{\mathrm{Boundary}}$ is determined as in §4.1, each boundary node is pulled in that direction with magnitude $F_{\mathrm{Boundary}}/(num\;\mathrm{Boundary}\;\mathrm{Nodes})$. All non-boundary wall-nodes are white, and cytoplasm nodes are not rendered. (*b*) Cells expanding out-of-plane (white) are chosen stochastically at simulation initiation and at the end of every cell cycle (details in §3.5). All other cells expand in-plane (red). (*c*) Cell growth directions are shown. Cells whose nodes are green (red) are preferentially expanding anticlinally (periclinally). Cells preferentially expanding out-of-plane or out-of-boundary grow with uniform mechanical properties along their wall (white). (*d*) Cell progress $CP_{i}$ (electronic supplementary material, section S1B) is shown for each cell. Cells with smaller $CP_{i}$ have recently finished a cell cycle, whereas cells with larger $CP_{i}$ are about to finish a cell cycle. (e) Visualization of all wall nodes in representative model simulations of both 2D and P3D simulations are visualized at t=40 h. Cell lines that were in L1 and L2 at time t=0 are shown with green wall nodes; all others are shown in white.

**Figure 4.**
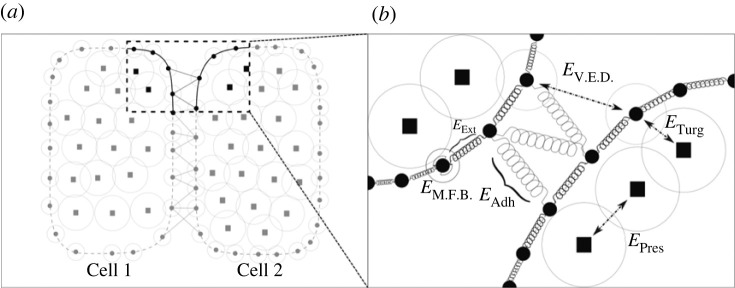
Diagram of the mechanical cellular and cell–cell interaction submodel. (*a*) Two interacting cells represented by heterogeneous collections of linked wall nodes (solid circles) and the cytoplasmic nodes (squares) with characteristic Morse potential ranges (rings). (*b*) Cell nodes interacting with each other through mechanical forces represented by potentials described in table 1.

3.2 $$\eta_{i}\frac{d}{dt}C_{i}^{j}=\;-\overset{M_{i}}{\underset{k=1}{\sum }}\nabla E_{\mathrm{Pres}}(C_{i}^{k},C_{i}^{j})-\overset{N_{i}}{\underset{k=1}{\sum }}\nabla E_{\mathrm{Turg}}(W_{i}^{k},C_{i}^{j}),$$

where $\eta_{i}$ is a cell's damping coefficient, which represents the relative viscosity experienced by the small amount of mass represented by each node as in [24]. Also as in [24], $\eta_{i}$ is increased by 10 times in the bottom layer of the simulated SAM, since that portion of the simulation boundary represents the SAM's interface with the more differentiated, less flexible tissue below the shoot apex and provides a barrier that allows the simulated SAM tissue to generate internal compression. Compressive forces are also passively provided by the non-dividing boundary cells on the sides of the simulated SAM. The force $F_{\mathrm{Boundary}}$ is applied to outward-facing nodes on the simulation boundary belonging to cells in the tunica (figure 3*a*). We implement this force to represent the stresses that the surrounding tissues apply to the tunica of the SAM, and a detailed description of this force and its justification is given in electronic supplementary material, section S1A. In particular, $F_{\mathrm{Boundary}}$ has a direction to promote experimentally observed tissue curvature and has an experimentally calibrated magnitude. Potentials *E* in the equations (3.1)–(3.2), yielding forces acting on nodes representing cell wall and cytoplasm, are described in table 1 and illustrated in figure 4. They have a form of a Morse or linear spring type potential for forces between nodes located at positions $x,y,z\;\in R^{2}$,

$$E_{\mathrm{Morse}}(x,y)=U_{\mathrm{Morse}}\exp\left(-\frac{{\left\|x-y\right\|}_{2}}{\xi_{\mathrm{Morse}}}\right)-V_{\mathrm{Morse}}\exp\left(-\frac{{\left\|x-y\right\|}_{2}}{\gamma_{\mathrm{Morse}}}\right),$$

$$E_{\mathrm{Linear}\;\mathrm{Spring}}(x,y)=\frac{1}{2}k_{\mathrm{Linear}\;\mathrm{Spring}}{\left(\|x-y{\|}_{2}-l_{\mathrm{Linear}\;\;\mathrm{Spring}}^{\mathrm{eq}}\right)}^{2}$$

$$\mathrm{and}\;E_{\mathrm{Bending}\;\mathrm{Spring}}(x,y,z)=\frac{1}{2}k_{\mathrm{Bending}\;\mathrm{Spring}}{\left(\theta_{∠yxz}-\theta_{\mathrm{Bending}\;\mathrm{Spring}}^{\mathrm{eq}}\right)}^{2}.$$

**Table 1.** Model potentials and associated physical phenomenon.

potential	type	cellular property
$E_{\mathrm{Turg}}$	Morse potential	turgor pressure
$E_{\mathrm{Ext}}$	linear spring	mechanical stiffness & extensibility
$E_{M.F.B.}$	bending spring	microfibril bending stiffness
$E_{V.E.D.}$	Morse potential	volume exclusion between different cells
$E_{\mathrm{Adh}}$	linear spring	cell–cell adhesion (fig. 5 of [24])
$E_{\mathrm{Pres}}$	Morse potential	cytoplasmic pressure

Note that $E_{\mathrm{Turg}}$ represents a coarse-grained force potential between the cell wall and the cytoplasm, and that $E_{\mathrm{Pres}}$ represents this force potential between cytoplasm nodes. Given that the resolution of coarse-graining of both wall and cytoplasm were performed independently in [24], force potentials representing molecular dynamics between nodes of different types and nodes of the same type were calibrated independently. These formulae describe soft-core potentials (i.e. the potential has finite value as x→y). Such methods are commonly used in molecular dynamics simulations [47]. With soft-core potentials, the volume exclusion forces never approach infinity and promote numerical stability. Even though this means that volume exclusion forces may be exceeded, and two cells may be pushed to overlap, such a phenomenon would require tremendous force, which is not observed with the parameter ranges used in our model simulations. These forms of the potentials are also differentiable and have critical points at x=y for our parameter values, which guarantees that $-\nabla E_{\mathrm{Morse}}(x,y)$ is continuous in the variable $\|x-y{\|}_{2}$.

### 3.3. Chemical distribution controls growth and division rate of cells

Cells in the model are assigned concentrations of signalling WUS ([WUS]) and CK ([CK]) using calibrated concentration distributions as in [24]. Namely, WUS controls cell life cycle length, which is used to determine the rate at which cytoplasm nodes are added to a growing cell.

In L1 and L2, values of [CK] are maintained at 0 due to a lack of its receptors. In deep-layers (i.e. L3 or deeper) CK and WUS concentrations are independently calculated using the expressions calibrated in [24],

$$\begin{array}{rl} {}[\mathrm{WUS}]= & {\left[\mathrm{WUS}\right]}_{0}\exp(-\mu_{\mathrm{WU}S}\cdot r_{\mathrm{WUS}});\\ {}[\mathrm{CK}]= & {\left[\mathrm{CK}\right]}_{0}\exp(-\mu_{\mathrm{CK}}\cdot r_{\mathrm{CK}}); \end{array}$$

where $r_{\mathrm{WUS}}$ and $r_{\mathrm{CK}}$ are the distances from the centroid of each cell to the ‘signal centres’, which we introduce as point-approximations of the spatial centres of the WUS and CK expression domains (electronic supplementary material, figure S3A). For details about signal centres and cell division cycles see electronic supplementary material, section S1B.

WUSCHEL and cytokinin values also influence the probability that cells anisotropically expand in-plane anticlinally or periclinally, respectively as in [12]. We also adopt the layer-specific cell division mechanism from [12] and apply it to determine the orientation of the division plane. When following that mechanism, cells in the apical corpus divide with respect to mechanical cues, and cells in the basal corpus orient their division plane stochastically based on a probability distribution parametrized by relative levels of WUS and CK. For details on the antagonistic roles of WUSCHEL and cytokinin, see electronic supplementary material, sections S1C and S1D.

### 3.4. Coupling submodels into a cell-based model

Computational implementation of the mechanical submodel of the SAM has the smallest time step among all submodels, with Δt≈0.4 s of represented time. The chemical division submodel is run every time a cell cycle completes, and the distributions of cell cycle lengths were experimentally calibrated in [24], and their implementation is discussed more in §3.3. We assume that division occurs at the end of each cell cycle as in [12]. Simulations were run to represent 40 h as in [10,24]. Coupling of the mechanical and chemical signalling submodels in space is achieved implicitly through the common use of the same spatial scale (i.e. the micrometre scale) and the ‘signal centre’ (electronic supplementary material, figure S3A), whose positioning is determined by the size of cells from the mechanical submodel. For the P3D submodel, horizontal displacement of cell centroids from the apex of the SAM was used to categorize each cell as a central or peripheral zone cell, which, along with the cell layer, determines the probability of that cell growth polarizing along or out of the plane.

### 3.5. Description of a pseudo-three-dimensional model

The apical surface of the SAM (perpendicular to the cross-sectional plane) has a radial symmetry (see §3.1), and so we assume in the P3D model that some cells are preferentially expanding perpendicular to our cross-section. We capture this in P3D model simulations by labelling the cells as having growth direction polarized out-of-plane (i.e. growth direction polarized perpendicular to the longitudinal-section) or in-plane (along the model cross-section), as in electronic supplementary material, figure S3E. Experimental data suggests that the fraction of cells whose growth direction is polarized out of a longitudinal plane is different in the central and peripheral zones. To quantify this, 450 cell divisions in wild-type SAMs were observed and the division planes were manually classified as either radial or tangential, and a frequency distribution was obtained for cells in the central and peripheral tunica and corpus (see §4.1 for details about experimental estimation of this frequency distribution). These frequencies were then used in P3D model simulations as a spatially heterogeneous probability distribution to choose which cells in each zone grow out-of-plane for their entire cell cycle. An example of a P3D simulation showing which cells are growing in- and out-of-plane is provided in figure 3*b* and electronic supplementary material, video S1.

In the P3D model, we also treat out-of-plane polarized cells as growing isotropically—i.e. we assign wall nodes of these cells uniform mechanical parameters, unlike those for the anisotropically expanding cells. Cells in the 3D biological system anisotropically expanding out-of-plane will have their longest axis orthogonal to the longitudinal section. We represent this in P3D by giving out-of-plane expanding cells a smaller longitudinal sectional area than those polarized along the model plane (approx. 25% smaller), since only their minor axis will be shown explicitly in simulations. When a cell growing out-of-plane completes its cell cycle, one daughter cell does not fall within the cross-section and is not represented explicitly in the simulation. Whenever an in-plane cell divides (§3.3), both of its daughter cells are assigned to have their growth polarized either in-plane or out-of-plane based on probability distributions as discussed in the next paragraph. Lastly, cells expanding out-of-plane have a terminal expansion size smaller than those in-plane (electronic supplementary material, section S1B).

## 4. Results

### 4.1. Model calibration

The following subsections describe the calibration of novel model components. For more details on the general model parameters (e.g. volume exclusion, adhesion, etc.) please see the electronic supplementary material, section S3. Moreover, electronic supplementary material, section S2 describes the initial conditions for all simulations and justification for their use. In [12], we performed sensitivity and perturbation analyses showing that these initial conditions did not introduce artefacts impacting the model results.

#### 4.1.1. Estimation of a boundary force magnitude

In [48], the SAM was studied under the assumption that it behaved as though it was a pressurized shell providing a justification to relate the tension-induced pressure directly to modified boundary conditions applied to the SAM. Electronic supplementary material, section S1A derives the following expression for the boundary conditions based on the pressure experienced by the SAM $P_{0}$, the average width of a cell *w*, and the radius of a sphere which approximates the SAM surface $r_{\mathrm{Ex}}$,

4.1 $$|F_{\mathrm{Boundary}}|=\frac{r_{\mathrm{Ex}}}{2}\cdot w\cdot P_{0}.$$

The radius of curvature $r_{\mathrm{Ex}}=80.1\;\mu m$ was chosen by selecting the L1 cells in 3D and fitting a sphere to them for 17 wild-type SAMs. The value of w=7.09 μm comes from measuring the diameter of each cell in the SAM in the direction normal to the longitudinal plane (see electronic supplementary material, section S5 for experimental and image analysis methods). The value of $P_{0}\in [0.66,0.98]\;\mathrm{MPa}$ was taken from the literature [48]. These values applied to equation (4.1) yield $|F_{\mathrm{Boundary}}|\in [190,280]\;\mu N$. When running simulations, we call 190 μN *‘*low’ force condition, 235 μN *‘*average’ force condition, and 280 μN *‘*high’ force condition. We examined the effects of running the simulations with inordinately large tension magnitudes using 560 μN which we refer to as the ‘2×’ force condition. If we set $|F_{\mathrm{Boundary}}|=0$, then we call this the free boundary condition.

#### 4.1.2. Calibration of the out-of-plane expansion frequency

To calibrate the frequency of cells growing out-of-plane, previously reported live time-lapse of plants expressing a fluorescent nuclear reporter (35S::H2B- mYFP) [49] were used to analyse cell division orientation in distinct zones of the SAM. Registration of time series of 512 × 512 × 20 images using Fijiyama on ImageJ was used to align the nuclear reporter construct across the time series imaged every 1–1.5 h. Image slices capturing the layer 1, layer 2, apical corpus and basal corpus were isolated manually in Adobe Photoshop. Superimposing two sequential time series allowed for the manual identification of the new nucleus after divisions.

These nuclei were manually classified to be aligned radially (both nucleus centres could be touched with a single radius line from the centre of the SAM) or to be tangentially aligned (electronic supplementary material, figure S5). Additionally, the nucleus within an eight-cell diameter across the centre was manually classified to be in the central zone (CZ) while cells outside this region were classified as divisions in the peripheral zone (PZ). This analysis gave us a region-specific calibration of the relative frequency of in-plane anticlinal divisions to out-of-plane divisions. It is important to note that in this method, it is difficult to accurately detect periclinal divisions, as the division planes may be parallel to and between z-stack slices. To remedy this, we also incorporated established frequencies of periclinal divisions to in-plane anticlinal divisions taken from our previous work [12]. The final values used to parametrize probabilities of cell growth polarization out of the plane in P3D enabled simulations is in table 2.

**Table 2.** Frequency of out-of-plane divisions by functional zone.

region	probability of out-of-plane growth polarization (%)
central zone tunica	47.4
central zone apical corpus	17.8
central zone basal corpus	10.5
peripheral zone tunica	43.4
peripheral zone apical corpus	29.0
peripheral zone basal corpus	18.2

### 4.2. Computational model predictions

#### 4.2.1. The biologically calibrated pseudo-three-dimensional model maintains the monolayer structure of the shoot apical meristem epidermis observed in experiments

**Figure 5.**
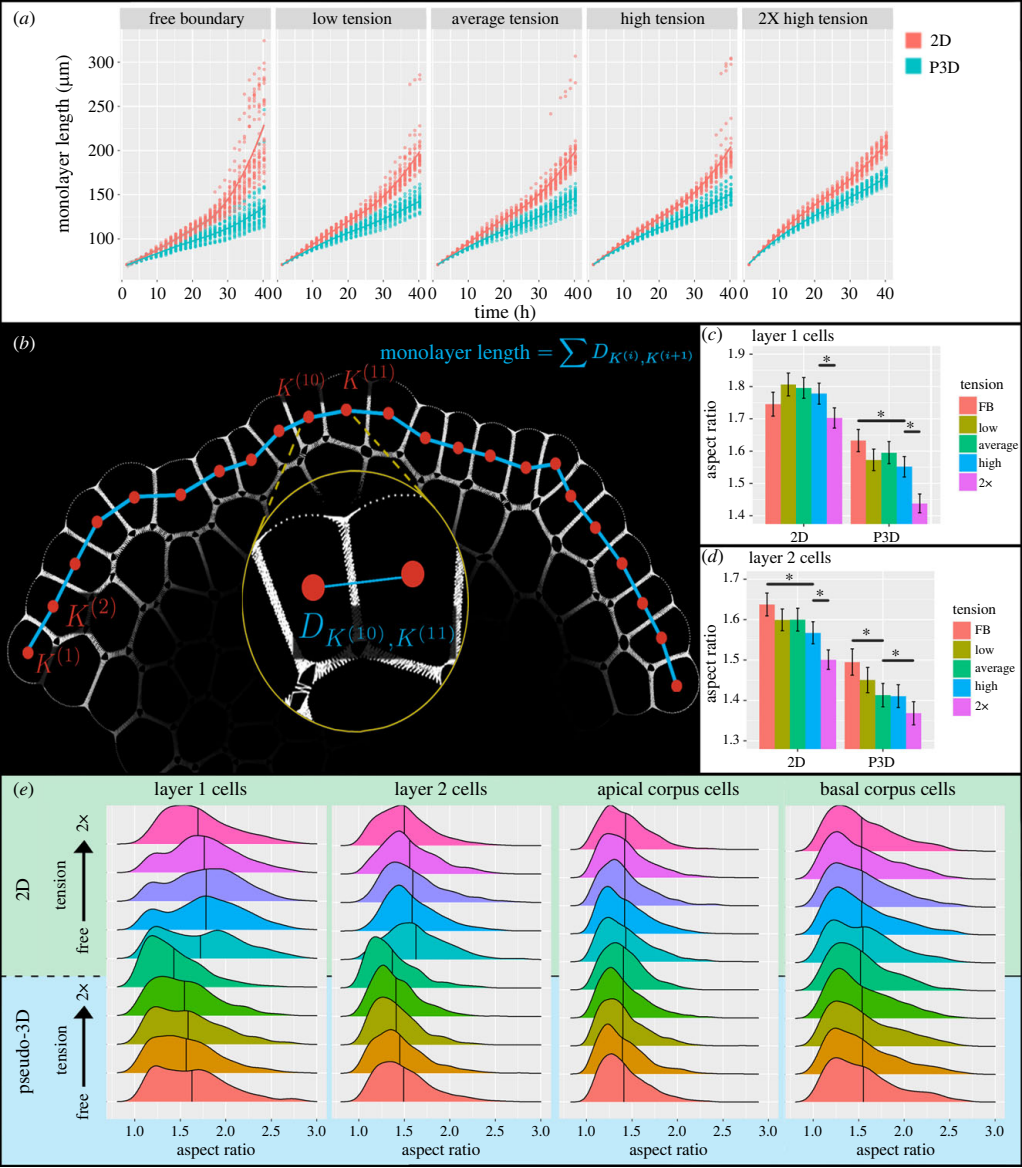
Apical surface structure. (*a*) Monolayer length of the epidermal layer of the SAM over time. Each panel shows longitudinal data from in-plane (red) and out-of-plane (blue) simulations under different tension ranges. Discontinuities are present in both 2D and P3D simulations under the free boundary condition, and only persist in the 2D simulations as tension increases. Jumps in the monolayer length are sufficient to indicate a break in L1 monolayer structure. (*b*) Schematic of the monolayer length. Details of this computation and its properties are in electronic supplementary material, section S4A. (*c,d*) Mean aspect ratios of both the 2D and P3D model cells in layer 1 (*c*) and layer 2 (*d*). Error bars are 95% confidence intervals computed with the Benjamini-Hochberg correction. Conditions with non-overlapping error bars (also indicated by *) have statistically significantly different means (*p* < 0.05). (*e*) Frequency distributions of the aspect ratio of cells in layer 1, layer 2, apical corpus and basal corpus. Each frequency distribution comprises cell aspect ratios from 30 simulated SAMs that were run to 40 h. In each column, the top five graphs (green region) are taken from 2D simulations, and the bottom five (blue region) are taken from P3D simulations. The five frequency distributions per region were generated under different levels of boundary tension, from free boundary to 2×. Rounded cells have aspect ratio 1, while elongated cells have higher aspect ratios. Cells in the 2D simulations are tightly squeezed by their neighbours, increasing their aspect ratios.

In wild-type SAMs, the epidermal and subepidermal layers of the SAM (collectively called tunica) are maintained as clonally distinct layers. Failure to maintain these layers in the wild-type SAM results in misplacement of organs. However, in 2D model simulations initialized with these layers, the tunica's layer-structure deteriorates within 40 h. In those 2D simulations, cells in the tunica tend to become highly compressed and elongated. This happens because cell divisions occurring too frequently in the same cross-section plane cause some cells to be pushed out of the layer and become connected with neighbouring cells above or below them. To quantify the proportion of SAMs whose layer structures cannot be maintained within 40 h, we introduce and observe the time evolution of *monolayer length* of the epidermal layer of model simulated SAMs (figure 5*a,b* and electronic supplementary material, section S4A for technical details), which evolves continuously in time if a monolayer of cells is maintained.

Of 30 2D model simulations at each tension level (as defined in §4.1), 43% of SAMs exhibit monolayer disruption in the free boundary condition and 7–10% in the low, average and high tension levels. Under the 2× tension condition, we did not capture any monolayer disruption in 2D model simulations. In P3D model simulations, only 7% of free-boundary SAMs exhibited monolayer disruption, and all samples maintained monolayer structure for any higher tension level. This suggests that cells anisotropically expanding out-of-plane offsets structurally disruptive cell–cell crowding in the direction of the longitudinal plane. The only quantifiable instances of monolayer breakdown in P3D simulations were under the free boundary condition, which demonstrates that there is a stabilizing effect of experimentally calibrated boundary forces on the layered tunica structure of the SAM.

#### 4.2.2. Tunica cell shape distribution is sensitive to cell–cell crowding without considering out-of-plane growth

To further examine the crowding of cells in the epidermal and subepidermal cell layers which may disrupt cell monolayer structure, we calculated the cell aspect ratio distributions in the L1 and L2 cell layers, the apical corpus and the basal corpus. The aspect ratio of a cell is defined as the ratio between cell length along its longest axis and its length in the perpendicular direction as in [12], and it is used as a measure of cell elongation. The mean aspect ratio of cells in the L1 and L2 is larger in 2D simulations than in P3D simulations for any tension level (figure 5*c–e*). The large aspect ratios obtained in the 2D simulations are symptomatic of individual cells being ‘squeezed’ into an elongated shape due to cell crowding in the same layer.

This is significantly reduced by out-of-plane expansion in the P3D model (Tukey honestly significant difference (HSD) test shows *p <* 10^−15^ for the L1 impact and *p <* 0*.*01 for the L2 impact). Moreover, L1 cells in the 2D model showed no significant differences in aspect ratio between any levels of tension that were tested pairwise, with the exception of the 2× boundary force condition. In the P3D model, increasing tension provides a significant decrease in the aspect ratios of tunica cells (figure 5*c,d*). This indicates that without accounting for 3D cell anisotropic expansion, the individual cell shape is dominated by cell–cell crowding. We neither observed nor expected substantial changes in the basal corpus, since the division plane orientation of cells in the basal corpus is determined by signal concentrations that were not perturbed in these tests.

**Figure 6.**
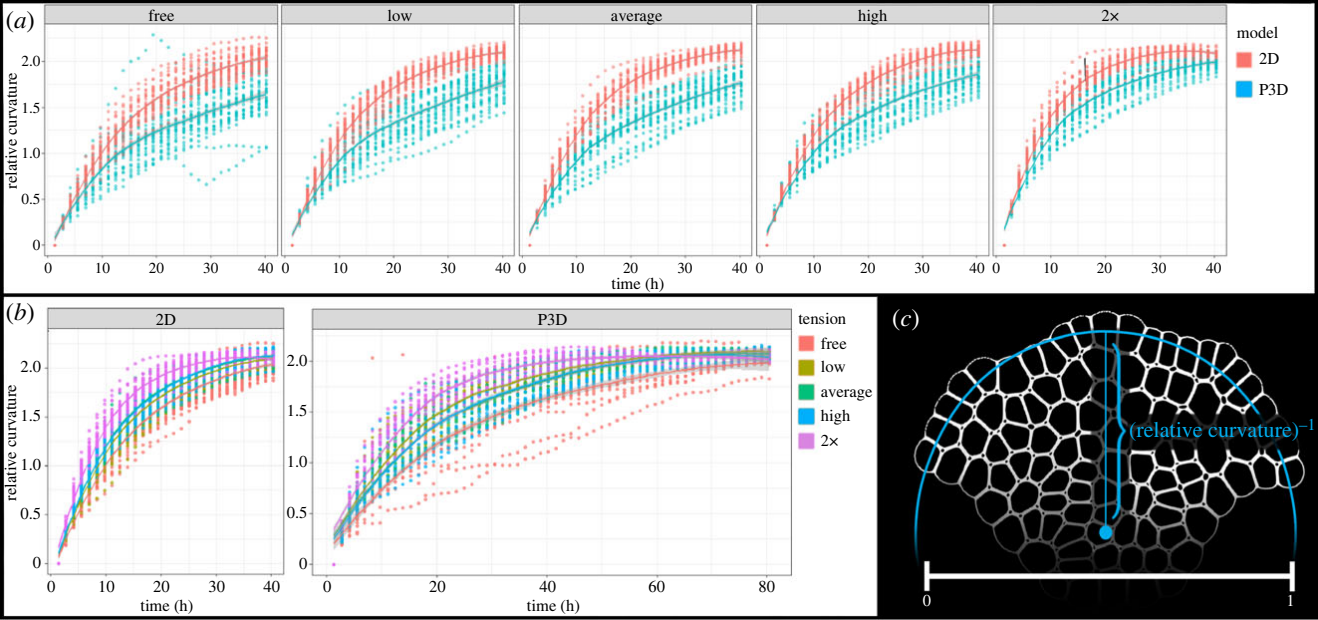
Tissue shape is robust to P3D or 2D model selection and choice of boundary force magnitude. (*a,b*) The relative curvature for 30 SAMs per tension level are shown for both 2D and P3D simulations. In each facet, the horizontal axis shows the timeline of the simulation. These data are shown in two ways, splitting data points between multiple graphs by (*a*) tension magnitude levels and (*b*) convergence of relative curvature in 2D and P3D simulations. In each graph, the vertical axis describes the curvature of the simulated SAM. Each facet shows the individual data points from 30 simulations, and the ribbons around each interpolated line show the standard error. Invisible or non-overlapping error ribbons for any point on the horizontal axis represent significant differences between the faceted populations at that time with *p* < 0.05. (*c*) Schematic of the calculation of relative curvature (details in electronic supplementary material, section S4B). To investigate shape while remaining agnostic to absolute size, the SAM is scaled down to width 1.

#### 4.2.3. Tissue shape and apical corpus cell shapes are robust to mechanical perturbation

We noticed that the impacts of model choice (2D versus P3D) and varying the magnitude of boundary forces on the distribution of cell aspect ratios were restricted to the tunica, with no significant differences in the corpus (figure 5*e*). We then sought to determine whether the tissue-scale shape was also robust to these factors. Tissue shape was quantified by introducing and calculating *relative curvature*, which captures the shape of the SAM while being agnostic to the absolute size of the simulated tissue (figure 6*c* for a diagram and electronic supplementary material, section S4B for formulation and details). We use this normalized measurement to analyse the level of being curved for the top surface of SAM. Comparison between the simulated non-normalized SAM and experimental SAM radii of curvature is in the electronic supplementary material, figure S7, showing a good consistency throughout the development. Figure 6 shows that most simulated SAMs from both the P3D and 2D models approach the same relative curvature value by 40 h. Simulated SAMs that do not reach the same relative curvature in 40 h were obtained in the P3D model, and this is due to the reason that lower in-plane division rate in the P3D model simulations results in a slower rate of convergence of P3D model simulated SAMs to their calibrated curvature.

Moreover, as the magnitude of boundary force increases, the variance of the relative curvature of the SAM decreases significantly in both the 2D and P3D models. Relative curvature variance decreases from 0.06 under the free boundary condition to 0*.*005 under the 2× force condition (§4.1). Figure 6*b* shows that increasing the magnitude of boundary force increases the rate at which the curvature of the SAM is established. However, it is noteworthy that mean relative curvatures of 2D and P3D free-boundary condition model simulated SAMs at 40 h is very similar to the simulated SAMs subjected to boundary forces (recalling that these forces act to restore the SAM curvature towards $r_{\mathrm{Ex}}$, described in electronic supplementary material, section S1A). These observations together with the robustness of apical corpus cell aspect ratios, suggest that there may be a regulator of cell and tissue shapes. We suspected that this regulation is an emergent property of mechanically determined division plane orientation in the apical corpus, motivating our investigation in the following sections.

#### 4.2.4. Two-dimensional and pseudo-three-dimensional models differ in apical corpus structure and division patterning yet produce robust tissue and cell shapes

We analysed division plane patterning via calculation of the percentage of periclinal divisions (measurement specifics in electronic supplementary material, section S4C). The division planes in 150 2D simulated SAMs' apical corpus divide at 52.1% periclinal frequency and in 150 P3D simulations divide with 47.5% periclinal frequency (two-way ANOVA, *p <* 0*.*002). Both 2D and P3D model simulation percentage-periclinal means are within the confidence bounds determined in our previous experimental study [12]. This change in in-plane division patterning occurred while cell and tissue shape remained largely unchanged, evident from the aspect ratio (figure 5) and relative curvature (figure 6).

**Figure 7.**
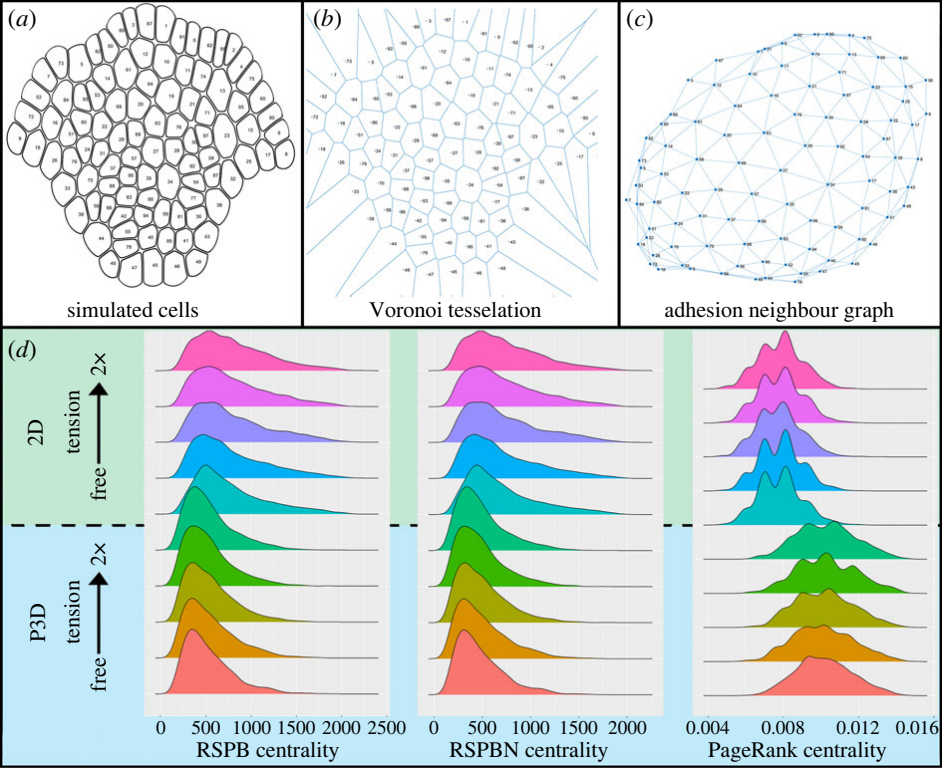
Impact of P3D behaviour on tissue structure. (*a*) Cell wall nodes from a simulated SAM are shown in blue. An arbitrary label is placed on its centroid. (*b,c*) A Voronoi tessellation (*b*) was performed on each centroid to extract the adhesion neighbour network (*c*). (*d*) Frequency distributions of RSPB random-walk centrality, RSPBN centrality and PageRank centrality of cells taken from simulated SAMs at t=40 h. Centrality values were pooled from 30 SAMs from each tension-level in both 2D (green region) and P3D (blue region).

In order to confirm that there was a distinct structural difference between the simulated tissues in the 2D and P3D models, we examined three more abstract quantifications of tissue structure patterning using the simulated SAM adhesion partner graph (figure 7*a–c*). This method has been used before for analysing many types of tissues [50]. *Centrality* has been used as a measure of how important an individual node in a graph (or cell in a tissue, in this case) is for communication [50,51]. This has been used in the SAM specifically to quantify the relative importance of any individual cell in diffusion-based signalling [5]. We chose to investigate the frequency distribution of three established types of centrality: *random shortest path betweenness centrality* (RSPB centrality) [52], *random shortest path betweenness net centrality* (RSPBN centrality) [52] and *PageRank centrality* [50].

The frequency distributions of the RSPB and RSPBN centralities of cells in 30 SAMs were shifted significantly lower between 2D and P3D simulations (figure 7; ANOVA test, *p <* 10^−15^ for RSPB centrality, *p <* 10^−16^ for RSPBN centrality). The PageRank centrality distribution of cells was significantly higher in P3D simulations (ANOVA *p <* 10^−15^). While applying these metrics to the model simulations representing a longitudinal section of a tissue (as opposed to the full 3D tissue *in vivo*) is rather abstract, it indicates substantial differences in the patterning between the 2D and P3D models in addition to the percentage periclinal division patterning. Despite the marked difference in tissue structure and division patterning, the tissue shape distributions (via relative curvature) and cell shape distributions (via aspect ratio) are robust to model selection.

Since, in general, it is recognized that plant tissue shape is a direct consequence of its structure and division patterning, and in both 2D and P3D simulations the apical corpus divides in response to mechanical cues, there may be some non-trivial property of the mechanically determined division plane placement mechanism that is related to the regulation of cell and tissue shape. Experimentally, this would be consistent with the fact that mutant SAMs, experiencing higher levels of CK (pCLV3::LhG4; 6xOP::ARR1-ΔDDK-GR) and also no CK (cytokinin receptor mutants), have substantially different shapes and division patterning [12]. In (pCLV3::LhG4; 6xOP::ARR1-ΔDDK-GR) mutants, the characteristic shape of the dome is observed to be qualitatively taller and more pointed and, on the cell scale, a clear deviation from wild-type division patterning is observed (figure 4 from [12] for a representative). In *wus-1* mutants, division patterning also deviates to promote periclinal division, and the *wus-1* mutant meristem is notably flatter.

#### 4.2.5. Apical corpus structure is robust to perturbations of tunica structure

The percentage of periclinal divisions in the apical corpus in 2D and P3D model simulations was robust to variation of tension magnitude up to and including double the biological maximum value (two-way ANOVA, *p >* 0*.*1). This means that even under substantial mechanical perturbation of the tunica, the mechanically driven division patterning of the rib meristem remains unaffected. Values of RSPB, RSPBN and PageRank centrality throughout the tissue were also unaffected by tension in the tissue, indicating a robustness of SAM's tissue structure to boundary tension.

The tunica cells are tightly adhered to the apical corpus cells, it is noteworthy that even under the 2× tension condition, there was no impact of the boundary forces on the shape or structure. This is in spite of the fact that the model apical corpus cells divide in response to local mechanical cues. This suggests that there is some other emergent phenomenon that prevents the effect of tension tangent to the SAM surface from propagating into the apical corpus structure.

## 5. Discussion

In this paper, a P3D cell-based SCE model of a longitudinal section of the SAM in *Arabidopsis thaliana* is developed and calibrated using 3D experimental imaging data. The P3D model is novel by taking into account anisotropic expansion of cells orthogonal to the longitudinal cross-section plane. 2D and P3D models were applied to study the impacts of epidermal tension within the biologically relevant range resulting from the connection of the SAM to the surrounding tissue, on the maintenance of SAM tissue shape and structure. In particular, the tunica layers, L1 and L2, both have a distinct layer structure, which is critical to properly place organs and produce different cell types during development. Maintenance of such layer structure requires that cell division follows a specific rule. The cell division plane placement mechanism introduced in [12] was shown to preserve this important morphology under epidermal tension in this study.

Model simulations demonstrated that cell shapes in the tunica were dependent on the magnitude of the SAM boundary tension in the P3D model, while other effects, such as increased cell–cell crowding, dominated tunica cell shapes in the 2D model. Nevertheless, cell and tissue shapes in the corpus were similar in both 2D and P3D model simulations. Upon analysis of division patterning and cell neighbourhood structure, it is shown that this may be due to the local stress-based division plane orientation mechanism. This was further supported by the observations that the model simulations with free boundary conditions produced tissue and corpus cell shapes very similar to those obtained in simulations with boundary forces that give rise to experimentally observed curvature.

Moreover, comparison between the 2D and P3D models simulations revealed regulatory functionality of the mechanically driven division plane mechanism in tissue patterning. Boundary tension was shown to play an essential role in maintaining the layered structure of the SAM tunica, though its importance is overshadowed by cell–cell crowding effects if 3D expansion is not considered in the model. Cell–cell crowding and monolayer disruption observed in 2D simulations suggest that coordinated anisotropic cell expansion along a plane can lead to tissue morphological changes similar to intestinal crypt formation [53], or other epithelial invagination processes [54–56].

It was also observed that even substantially increased boundary tension along the tunica did not impact corpus structure or distribution of cell shapes in either the 2D or P3D models. This suggests that the cell division patterning in the apical corpus has some robustness to variations in the magnitude of tension applied along the tunica. This robustness can facilitate plant growth, as it implies that the cellular structure of the rib meristem could be robust to mechanical perturbations, whether they are extrinsic (e.g. tissue damage) or intrinsic (e.g. expansin-induced changes to tunica mechanical properties).

The mechanism at play involves mechanotransduction, and we found that it preserves shape under various perturbations. If this mechanism is indeed at play, then failure of a plant to preserve its shape may be directly attributed to its inability to participate in mechanotransduction. Since this mechanism involves WUS and CK, it suggests that WUS and CK may play an important role in the plant's ability to detect or respond to mechanical stresses.

Finally, we hypothesize, based on obtained results, that ectopic activation of WUS and CK signalling can modify the cell identity, so that the division planes are no longer determined mechanically. Therefore, there are several different directions of future study suggested by the current work. If the mechanical signals generated by boundary forces applied to the tunica do not impact the apical corpus structure and organization, and if the tunica and apical corpus are tightly adhered, it would be important to study how the mechanical signals from the tunica are kept separate from the corpus. It will be also worthwhile to investigate how WUS and CK affect the ability of cells in the corpus to receive mechanical signals. It is possible that there is a mechanical signalling pathway that WUS and CK regulate non-trivially, or there are signals independent of the ability of cells to respond to their local mechanical stresses. Identifying the downstream components of cell growth and division regulated by WUS and CK, and analysis of the cytoskeletal response to perturbation of these regulators may provide new insight about the maintenance of tissue structure.

Our model of the SAM has been calibrated in prior work [12,24] on a cellular and a subcellular scale using all available experimental data and, other than the P3D/2D and tunica boundary stresses presented in this work, there are no tissue-scale conditions applied to model SAM. Resultant structure of our model simulations is an emergent property of individual cells and subcellular components interacting with one another. Our previous work showed that a similar emerging structure is present in experimental SAMs, but experimental data on division patterning in the deep layers are presently not available to compare with the simulation results. This can be a future work to verify the modelling prediction.

In the future, we plan to build on the recently obtained results on the maintenance of the WUS protein gradient by *CLAVATA3* signalling [3] and the WUS concentration-dependent regulation of *CLAVATA3* transcription [4] to implement a dynamic signalling variant of the present model, wherein the WUS and CK gradients influence the mechanical model, and the mechanical model provides dynamically evolving domain for signalling submodel. We also plan to further refine and investigate the forces influencing the mechanical compression and deformation of cells in the deep layers by calibrating and implementing an inward compressive force on the corpus from the peripheral tissue as data becomes available. Recently it has been noticed that cells in SAM have cell walls with different thickness, which may give rise to non-uniform cell mechanical properties within the tissue. As future research, we will improve our model by calibrating parameters using experimentally measured layer-specific mechanical properties as these data become available and study how this will affect the shape formation of SAM. Another potential application of our model is to include cells in primordia as well as those between CZ and primordia to investigate how new organs are developed in SAM. Our current model only includes stem cells in CZ. Cells located in primordia become differentiated and experience more complicated and diverse dynamics. In particular, they maintain the layer structure while buckling occurs to give rise to new organs. Our model can be calibrated using experimental data for cells in primordia and applied to understand the role of both mechanical and chemical signals during that process.

Future experimentation including analysis of deep-layer division patterning under the conditions of mechanically perturbed SAM tunica tension, can show the independence between the corpus and tunica of the SAM predicted by the model simulations described in this paper. The spatial localization of this patterning in a functional zone would motivate the investigation of the functionality of this regulation. It may also suggest a self-maintenance programming of the SAM in order to maintain a consistent spatial domain and further to facilitate the characteristic phyllotactic patterning in the plant.

## Data Availability

Codes for the pseudo-3D and 2D models can be found from the GitHub repository: https://github.com/ICQMB/Study-of-the-Impacts-of-Turgor-pressure-Induced-Boundary-Tension-on-the-Maintenance-of-the-SAM. The data are provided in electronic supplementary material [57].
